# Stapled Repair of Benign Acquired Tracheoesophageal Fistula: Description of Novel Technique and Assessment of Outcomes

**DOI:** 10.7759/cureus.9854

**Published:** 2020-08-18

**Authors:** Ram Prakash Gurram, Senthil Gnanasekaran, Raja Kalayarasan, Pottakkat Biju, Chandrasekar Sandip

**Affiliations:** 1 Surgical Gastroenterology, Jawaharlal Institute of Postgraduate Medical Education and Research, Puducherry, IND

**Keywords:** tracheoesophageal fistula, benign acquired tracheoesophageal fistula, tef, sternocleidomastoid muscle flap, stapled dismantling, lateral approach, prolonged mechanical ventilation

## Abstract

Compared to less invasive measures, surgical repair is the most effective modality for managing benign acquired tracheoesophageal fistula (TEF). Traditionally, this involves dismantling of the fistula and suture repair of tracheal and esophageal defects using a lateral or direct approach. However, the best approach remains a subject of debate. We describe a novel and simple technique for dismantling a benign acquired TEF with the use of an endo-stapler and interposition with sternocleidomastoid (SCM) muscle flap. Eleven TEF patients underwent repair using this stapled repair technique. Retrospectively, the perioperative and long-term outcomes were assessed in these patients. There were no cases of procedure-related mortality or airway-related complications. Two patients developed transient vocal cord palsy and one developed esophageal leak. At a mean follow-up of 21.4 months, no fistula recurrence, dysphagia, or tracheal stenosis was observed. Hence stapled dismantling and SCM muscle interposition is a simple and safe technique for repair of benign acquired TEF.

## Introduction

Acquired non-malignant tracheoesophageal fistula (TEF) is a rare condition but can have disastrous consequences if not addressed appropriately. Its complexity demands critical clinical evaluation and meticulous management for optimal outcomes.

While most commonly related to prolonged mechanical ventilation (MV), other etiologies, such as trauma and surgery, also contribute to the development of this complication [1]. Among factors that can contribute to MV induced TEF, cuff pressure was the most important [2]. The use of high-volume low-pressure cuffs was not as detrimental as their counterparts (low volume high-pressure cuffs) in impeding tracheal mucosal blood flow and can potentially reduce the incidence of the complication. Still, such cuffs are not completely complication-free as they can be overinflated, compromising mucosal blood flow [3]. 

With an ever-increasing number of patients requiring prolonged MV, the burden of TEF is significant [4]. While this catastrophic complication can be managed by surgical therapy, surgery is not always straightforward; with various approaches and techniques available, surgeons must choose the intervention that best suits the patient’s specific condition. Two different approaches were described: the anterior approach and the lateral approach [5,6]. Each has its advantages, disadvantages, and limitations [7]. We describe our novel surgical technique, a form of lateral approach for TEF repair with technical modification.

## Technical report

Patients and methods

A search of prospectively maintained data from June 2015 to January 2020 was conducted for cases of acquired benign aerodigestive tract fistula managed within our institute’s surgical gastroenterology department. Retrospective analysis revealed 13 such patients who underwent surgical therapy as part of definitive management; among them, 11 patients had TEF and two had bronchopleural fistula. The 11 patients who planned to undergo/underwent surgical therapy with our technical modification were traced and analyzed (Table 1).

**Table 1:** Master chart depicting patient characteristics TEF: Tracheoesophageal fistula; CECT - Contrast enhanced computed tomography; RLN – Recurrent laryngeal nerve; SSI – Surgical site infection; LRTI – Lower respiratory tract infection; POD – post operative day

Sl no	Age(yr)/Sex	Cause of TEF	Tracheostomy	Interval between detection of TEF and TEF repair	Feeding access	Length of fistula in CECT (in cm)	Complications	Able to tolerate orals after how many days of surgery
1	53/M	Trauma	Yes	16 days	Nasogastric	2.4	RLN palsy, SSI	12 weeks
2	30/M	Prolonged intubation	Yes	4 months	PEG	3	-	POD7
3	26/M	Trauma	No	2 months	Jejunostomy	2.1	-	POD8
4	24/F	Prolonged intubation	Yes	6 months	Jejunostomy	2.4	Graft hematoma	POD10
5	18/M	Prolonged intubation	Yes	3 months	Nasogastric	2.7	Esophageal leak	16 weeks
6	52/M	Trauma	Yes	26 days	Jejunostomy	2.4	-	POD7
7	31/M	Prolonged intubation	Yes	4 months	PEG	4.2	-	POD7
8	27/M	Prolonged intubation	No	7 months	Jejunostomy	2.1	SSI, LRTI	POD12
9	23/F	Prolonged intubation	Yes	2 months	Jejunostomy	2.4	-	POD8
10	18/M	Prolonged intubation	Yes	3 months	Jejunostomy	1.8	LRTI, SSI	POD10
11	25/F	Prolonged intubation	Yes	3 months	Jejunostomy	2.1	RLN palsy, LRTI, SSI	14 weeks

Patient characteristics

Our 11 surgical patients included eight men and three women. The mean age at presentation was 29.7 years (18-53 years). The most common etiology was MV-related TEF, accounting for nine patients, whereas penetrating trauma was the inciting factor in two cases. 10 patients were initially managed elsewhere and referred to our center for definitive surgical therapy. Nine patients underwent a preliminary feeding procedure, two underwent percutaneous gastrostomy, seven underwent feeding jejunostomy, and two were managed with nasogastric tube feeds. We routinely perform feeding jejunostomy in all TEF patients. Tracheostomy was present in nine cases at the time of referral to our department.

**Figure 1:**
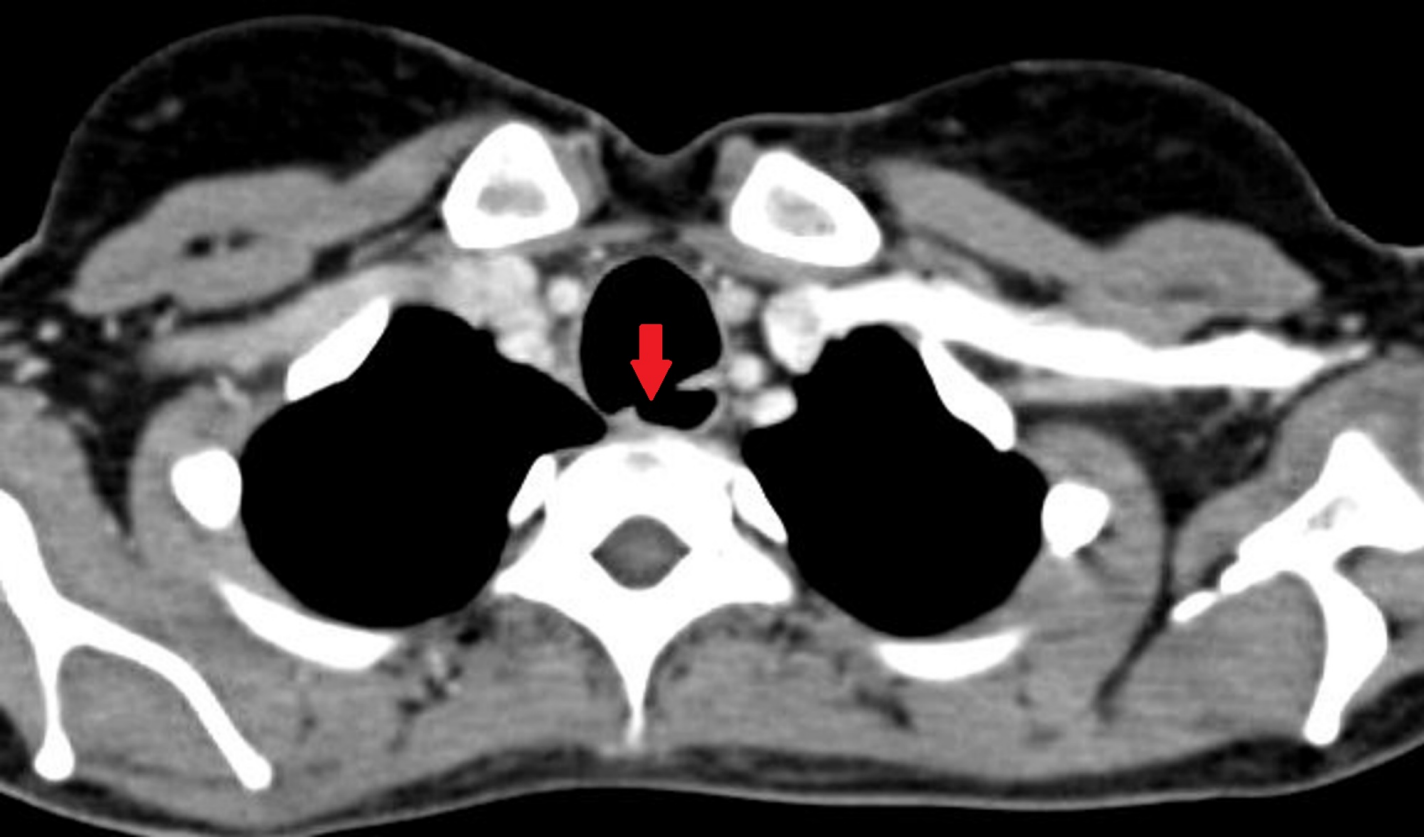
CECT demonstrating tracheoesophageal fistula (arrow highlighting fistula) CECT - Contrast-enhanced computerized tomography

Prior to definitive surgical therapy, flexible bronchoscopy was performed in all patients, mainly to assess any tracheal stenosis at or distal to the fistula level and to look for granulation tissue or ulceration around the fistula site. None of our patients showed evidence of tracheal stenosis distal to the level of fistula. All patients underwent contrast-enhanced computed tomography (CECT) of neck and thorax, as well as high-resolution computed tomography (HRCT) for the exact localization of fistula in relation to vertebral body level and to measure fistula length and diameter (Figure 1).

Mean fistula length was 2.51 cm (1.8-4.2 cm) and mean diameter was 0.78 cm (0.5-1.5 cm). HRCT also helps in gauging the extent of pneumonitis and subtle consolidation changes that can be missed with routine chest radiography; six patients showed lung consolidation changes in HRCT. Upper gastrointestinal endoscopy (UGIE) was routinely performed for all patients to ascertain the number of fistula, fistula location, and to look for ulceration around the fistula site. The mean distance of the fistula in UGIE was 17.2 cm (16-19 cm) from the incisors. Two referred patients had oral contrast study at presentation; otherwise, it was not routine for TEF diagnosis and was performed only in patients for whom CECT and bronchoscopy failed to definitively identify TEF in the context of typical symptoms.

Preoperative optimization

With communication between the aero-digestive tract and inevitable seepage of enteric contents into the respiratory tract, pulmonary infection is foreseeable. Patients were nursed in semi-recumbent position. The chest was optimized with aggressive physiotherapy and frequent lung toileting. Six patients required preoperative culture (sputum) based antibiotic therapy for the treatment of respiratory ailments.

Nutrition is another aspect requiring special care in this group of patients. As most patients were nutritionally suboptimal, dietician consultation was done for all patients and adequate input was ensured.

Once free of active infection, adequately optimized, and upon providing consent, patients were taken for definitive surgical management. The mean interval between diagnosis and surgical therapy was 86 days (16-192 days). This period of optimization also ensures that any friability and inflammation around the fistula site to settle. None of our patients were on MV at the time of surgery.

Anesthesia

The procedure was performed under general anesthesia. All patients were reintubated despite the presence of tracheostomy. With difficult intubation anticipated, fiber optic bronchoscopy was employed in all cases to prevent the ET tube from entering the fistula or creating a blind tract. Bronchoscopy also assured that the ET tube cuff was positioned beyond the fistula level. Video laryngoscopy guided intubation can also be used. Once adequate tidal volume delivery was ensured and airway pressures were maintained, the surgical procedure began. The patient was positioned supine with neck extended and head tilted to the right. Sandbags placed below the shoulder blades help maintain adequate neck extension.

Surgical procedure

Baisi et al. advocated a more conservative approach for the management of acquired non-malignant TEF repair for a select group of patients, avoiding major resection [8]. We describe our novel modification of the conservative surgical technique under the following steps:

1. Dissection and delineation of fistula

2. Fistula takedown with the stapler

3. Interposition of sternocleidomastoid (SCM) flap

 

Dissection and Delineation of Fistula

**Figure 2:**
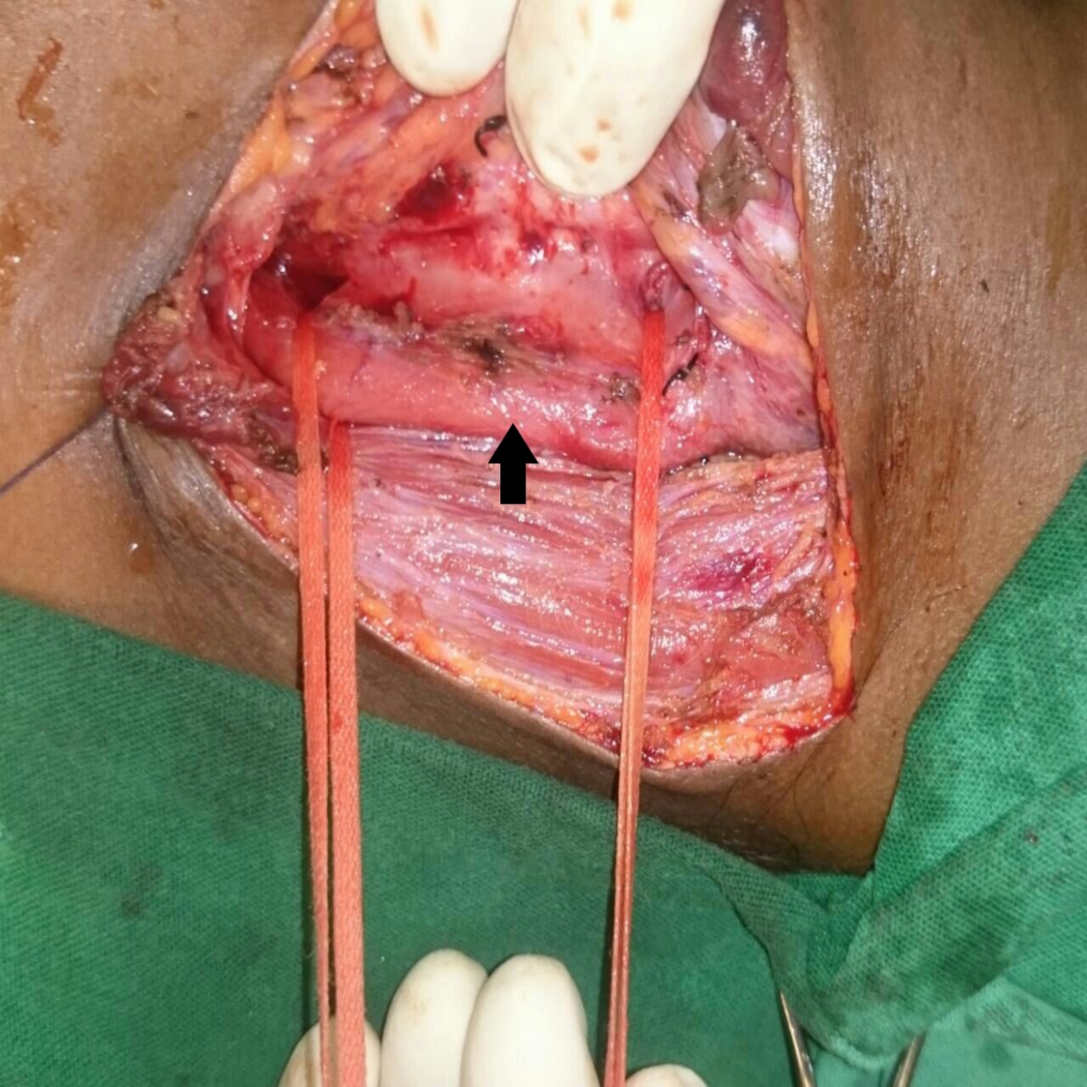
Intraoperative picture showing tracheoesophageal fistula and esophagus (arrow) looped by umbilical tape both proximal and distal to the fistula

An 8 to 10 cm left cervical incision was made along the anterior border of the SCM muscle. The incision was deepened to include platysma and fascia along the SCM muscle, and the muscle was retracted laterally. Lateral retraction of the SCM and division of omohyoid exposes the carotid sheath along with its contents. If present, the middle thyroid vein was identified and securely ligated. The inferior thyroid artery was also identified and securely ligated. A thyroid stitch was made to facilitate the removal of the left lobe of the thyroid from the field. If a nasogastric (NG) tube was not in place, one was passed through at this time. Preferably, a large-bore NG tube (18 French) was inserted as it not only helps with the identification of the esophagus but ensures adequate lumen within the esophagus. The larynx and trachea were retracted laterally with the assistant’s fingers, exposing the tracheoesophageal groove, and the recurrent laryngeal nerve was identified and preserved. Blind retraction with metal retractors in the tracheoesophageal groove is not advised as it can damage the recurrent laryngeal nerve. The esophagus was dissected and retracted laterally, opening the tracheoesophageal groove and facilitating fistula delineation. Dissection of the esophagus should be performed both proximal and distal to the fistula level to facilitate adequate looping of the esophagus, which helps provide more effective lateral traction as shown in Figure 2.

Further dissection was done in the groove to carefully outline the fistula without injuring the nerve. Once dissection was complete, the fistula was also looped with umbilical tape.

Fistula Takedown With Stapler

**Figure 3:**
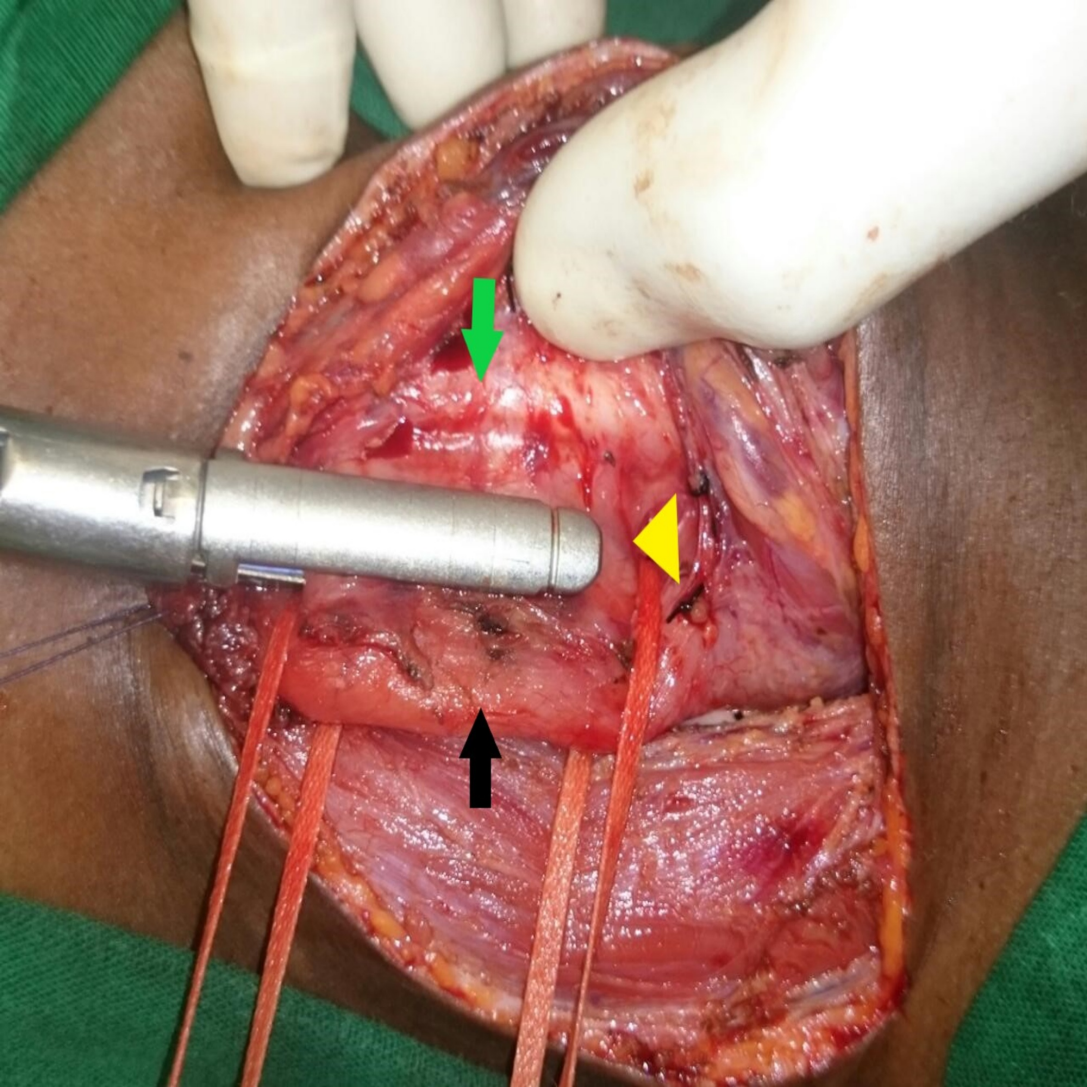
Laparoscopic linear stapler being used to divide the fistula Black arrow – oesophagus, green arrow – trachea, yellow arrow head- fistula incorporated into stapler.

Once the fistula was completely delineated and looped around, a 35mm laparoscopic linear cutter (Endopath™, Ethicon Endo-surgery, Cincinnati, OH, USA or Endo GIA™ 30 mm Reload with Tri-Staple™ Technology, Medtronic, Covidien products, Minneapolis, MN, USA ) was passed and its jaws were approximated around the fistula site (Figure 3).

**Figure 4:**
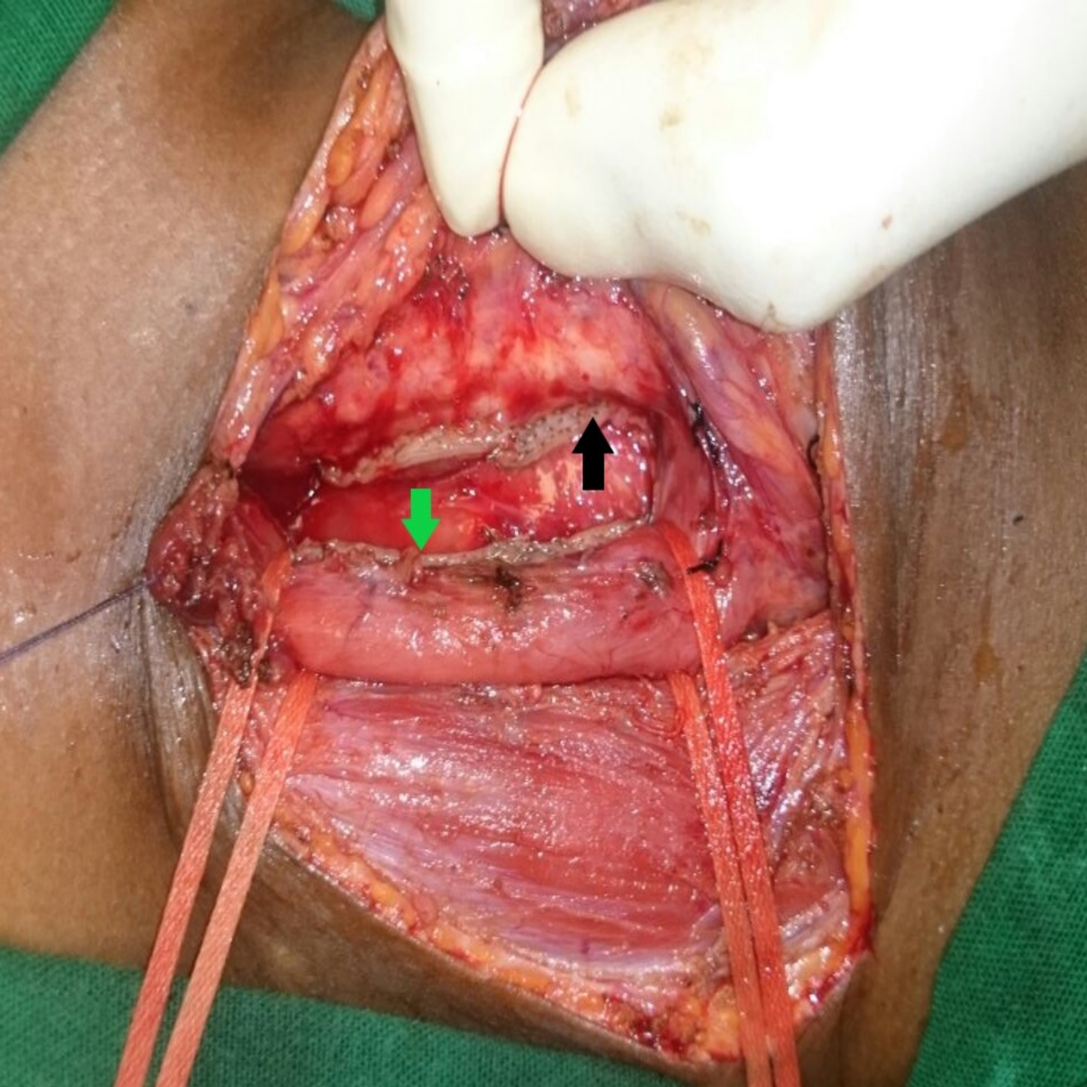
Staple lines after completer division of fistula Green arrow- esophageal staple line, Black arrow- tracheal staple line

Lateral retraction of the looped esophagus and gentle countertraction over the trachea helped create adequate space for safe stapler application. Before firing the stapler, the ET tube cuff was partially deflated to prevent cuff injury; free movement of the NG tube should also be ensured. The stapler was fired down through the looped and demarcated fistula site. If the fistula was not fully resolved with one stapler fire, the procedure was repeated with another stapler fired from the opposite direction on the other end. Once firing was done and the trachea and esophagus were separated, both stapled ends were inspected for integrity and defects (Figure 4).

No additional sutures were made on either end.

Interposition of Sternocleidomastoid Flap

**Figure 5:**
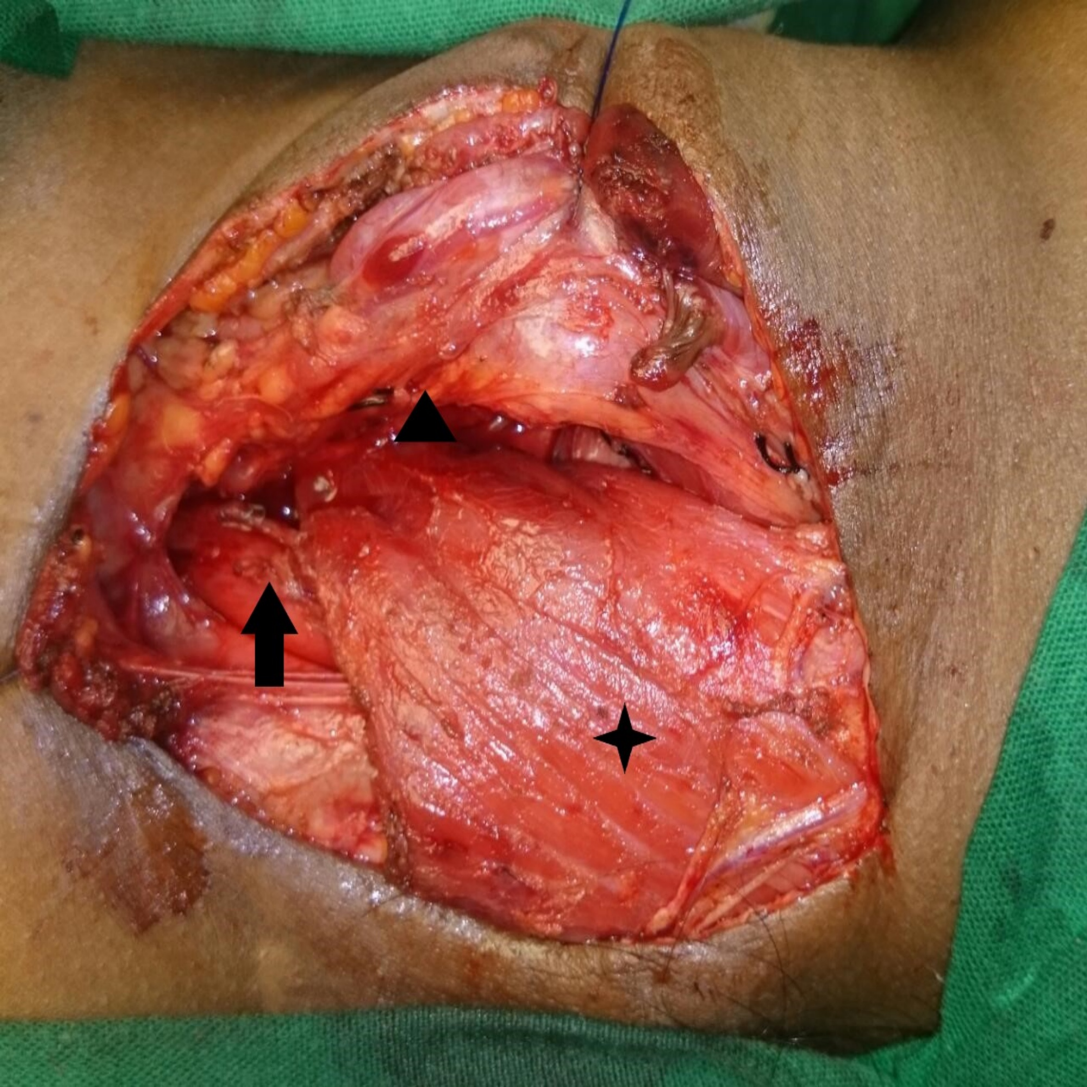
Sternocleidomastoid muscle flap (highlighted by star) is fixed to prevertebral fascia between trachea (highlighted by arrow head) and oesophagus (highlighted by arrow)

Segmental arterial blood supply of the sternocleidomastoid (SCM) enables its use as a pedicled graft [9]. It can be used as an inferiorly or superiorly based flap, depending on the region of interest where its purpose is to be served. It must be cut low in the neck for high cervical fistula and high in the neck for low cervical fistula [8]. For the inferiorly based flap, the muscle was divided between upper one-third and middle one-third after taking down the occipital artery. For the superiorly based flap, fascia of lesser supraclavicular fossa between two heads of the SCM was dissected and both sternal and clavicular heads were detached from their origin. It is important to avoid injuring branches of the ascending cervical artery as this will compromise the blood supply to the graft. The middle third part of the muscle, which is supplied by the superior thyroid and ascending cervical arteries, was rotated and used to interpose the gap between staple lines over the esophagus and trachea after flap creation. After checking for its easy extrusion between the trachea and esophagus, the muscle was sutured to the prevertebral fascia on the contralateral side with 4-0 Vicryl (Figure 5).

If the area needing to be interposed exceeded available width from the muscle, bilateral SCM flap was utilized. A 14 French suction drain was left in place and skin was approximated with staplers without separate closure of the platysma.

Results 

Out of 11 patients, this procedure was successfully completed in all but one who required tracheal repair, transhiatal esophagectomy, retrosternal gastric pull-up, cervical esophagogastric anastomosis. In this patient, the lower end of the fistula was at the T2 vertebral level; there was difficulty in looping the fistula, and in the process of looping around the fistula site, a longitudinal esophagus tear occurred, necessitating a change in procedure.

The mean operating time was 141 minutes (110-230 min) and mean blood loss was 85 ml (50-250 ml). The mean length of fistula measured intraoperatively was 2.76 cm (1.5-4 cm), correlating well with preoperative estimation via CECT (2.38 cm). In all cases, the SCM muscle was used as an interposition flap; it was used as a superiorly based flap in six cases and inferiorly based flap in three cases. In one case, bilateral SCM flap was used, while no graft was used in the case involving gastric pull-up.

All patients were extubated on the table after surgery and moved to the ICU. If the patient had a tracheostomy done preoperatively, it was reinserted following extubation, taking care to avoid mechanical disruption of the staple line. Patients ambulated on the same day and no perioperative deep venous thrombosis prophylactic medication was administered. Antibiotics were discontinued 24 hours after surgery if there was no evidence of respiratory sepsis. Three patients had lower respiratory tract infections requiring antibiotic therapy. If the patient had previously been on jejunostomy or gastrostomy feed, it was resumed on the day of surgery.

Postoperatively, there were no complications related to the tracheobronchial tract. One patient developed esophageal leak that resolved with conservative management. One patient developed graft hematoma, requiring evacuation, but the graft remained viable. While two patients developed transient left vocal cord palsy, this resolved in the follow-up period. Four patients developed superficial surgical site infection.

**Figure 6:**
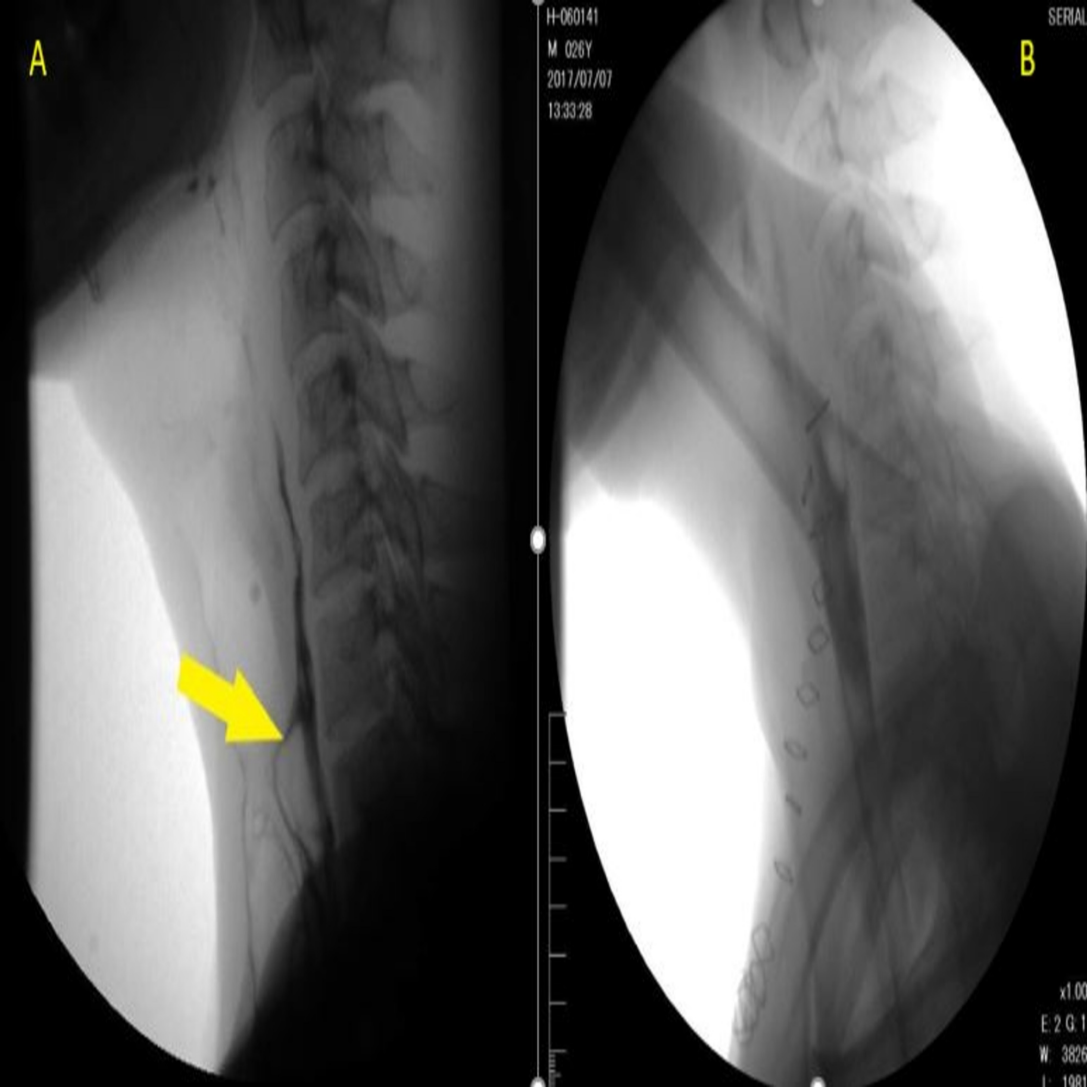
A) Preoperative oral contrast study showing fistula(arrow) B) Post-operative oral contrast study of same patient showing no evidence of either fistula or leak

Unless there were signs of an esophageal leak, patients underwent an oral contrast study with Gastrograffin on postoperative day 7 (Figure 6).

If the contrast study showed no leak, the patient was started on an oral diet. Out of 10 patients, nine underwent contrast study; one patient with esophageal leak was deferred. Among the nine patients, none showed persistent fistula or leak and, subsequently, were started on an oral diet. Seven patients were able to tolerate an oral diet at the time of discharge. The two patients with vocal cord palsy exhibited features of aspiration following oral diet initiation; as such, orals were withheld. They underwent post-operative swallow therapy and tolerated an oral diet in follow-up. One patient who developed esophageal leak was managed conservatively and discharged on jejunostomy feeds. He developed esophageal stricture, requiring postoperative endoscopic dilatation; following this, he was started on an oral diet. The mean duration of postoperative stay was 9.8 days (7-20 days).

There was no mortality in the immediate postoperative period or during the next six months of follow-up. The mean follow-up duration was 21.4 months (5-36 months). All patients tolerated oral diet at follow-up and there were no cases of recurrences. All patients were decannulated from tracheostomy after two months as per institutional protocol.

## Discussion

Unlike its malignant counterpart, surgery is the most effective treatment for benign TEF and should aim at a complete cure without significant morbidity. Surgery timing often determines the TEF repair outcome. Elective TEF repair after a decrease in the surrounding inflammatory response and adequate optimization provides optimal results. However, if significant aspiration is unavoidable - as in unconscious patients - a cervical esophagostomy, with or without decompressive gastrostomy, was advocated in the emergency setting. In other patients, preoperative optimization can be achieved by simple spitting of saliva and feeding by NG tube, nasojejunal tube, or feeding jejunostomy [10].

Postintubation TEF usually involves the cervical or upper thoracic esophagus; hence, it can be easily approached from the left neck. Tracheal resection and anastomosis with primary esophageal closure was recommended as the preferred procedure in some series while a more conservative approach of the simple repair was advocated in others [7]. However, tracheal resection and anastomosis are associated with increased morbidity and should be reserved for patients with concomitant tracheal stenosis or patients with extensive damage to the posterior tracheal wall. Tracheal stenosis at or distal to the fistula usually arises from chondritis and cartilaginous necrosis, resulting in granulation tissue formation that ultimately heals with fibrosis and circumferential stenosis. Pooled secretions, superadded infection, and gastroesophageal reflux disease may further add to the insult [11]. Older age, female sex, and prolonged intubation were considered high risk for tracheal stenosis [12]. None of our patients had distal tracheal stenosis; this may be due to the fact that our series included younger and primarily male patients, with most having a tracheostomy at the early stage after TEF diagnosis. Fistula dismantling and repair of both organs was usually recommended in the absence of tracheal stricture or stenosis [13]. Traditionally, the esophagus was closed in two layers and the trachea in a single layer. This suturing process was cumbersome and especially challenging in cases of posteriorly located TEF. This critical step of suturing was made relatively simpler in our technique with the use of the stapling device. The concept was derived from its successful use in various enteroenteric, enterovesical fistulas, and congenital TEF [14]. Endostapler use for benign acquired TEF repair in adults has not been previously reported. The three layers of staples in the laparoscopic linear cutter effectively seal both the tracheal and esophageal side, ensuring the integrity of both structures. While tracheal or esophageal narrowing may be a concern, meticulous dissection to isolate the fistula and looping the esophagus with the umbilical tape provided adequate space for stapler application, protecting vital structures from the firing line. Moreover, the smaller width of the laparoscopic linear cutter compared to open linear cutters facilitates its use for TEF repair. The technique described in this report is ideal for short TEF (<4 cm) in the absence of tracheal narrowing.

Mortality and morbidity outcomes with our surgical technique were satisfactory and comparable to existing literature [7,8,13,15]. Mortality in the various series range between 0-33%, whereas our series saw no mortality. Similarly, the fistula healing rate and appliance-free trachea were achieved in about 33-100% across the literature; this was 100% in our series at a six-month follow-up [7]. In the immediate postoperative period, 90% of our patients had an excellent outcome regarding fistula repair, except for one patient who developed esophageal leak. Despite the identification of the recurrent laryngeal nerve in all patients, two patients developed transient vocal cord palsy, likely due to neuropraxia or cautery injury; both patients recovered over a period of 12-18 weeks. Incidence of TEF recurrence following repair is reported as high as 11%, depending on underlying factors [1]. At a mean follow-up of 21.4 months, none of our patients experienced fistula recurrence or tracheal stenosis, further reinforcing the procedure’s safety.

## Conclusions

For repair of benign acquired TEF, stapled dismantling with SCM flap interposition is a feasible, safe, and easily reproducible technique with less short-term morbidity. This repair technique can be done electively in fistulas measuring less than 4 cm in length and involving the upper trachea without associated tracheal stenosis. A prospective study with a larger population is needed to further validate the long-term outcomes of this procedure.
